# On-Demand Generation of Entangled Photon Pairs in the Telecom C-Band with InAs Quantum Dots

**DOI:** 10.1021/acsphotonics.1c00504

**Published:** 2021-07-15

**Authors:** Katharina D. Zeuner, Klaus D. Jöns, Lucas Schweickert, Carl Reuterskiöld Hedlund, Carlos Nuñez Lobato, Thomas Lettner, Kai Wang, Samuel Gyger, Eva Schöll, Stephan Steinhauer, Mattias Hammar, Val Zwiller

**Affiliations:** † Department of Applied Physics, Royal Institute of Technology, Albanova University Centre, Roslagstullsbacken 21, 106 91 Stockholm, Sweden; ‡ Department of Electrical Engineering, Royal Institute of Technology, Electrum 229, 164 40 Kista, Sweden

**Keywords:** semiconductor quantum dots, telecom wavelengths, entangled photons, two-photon resonant excitation, single-photon source, quantum state tomography

## Abstract

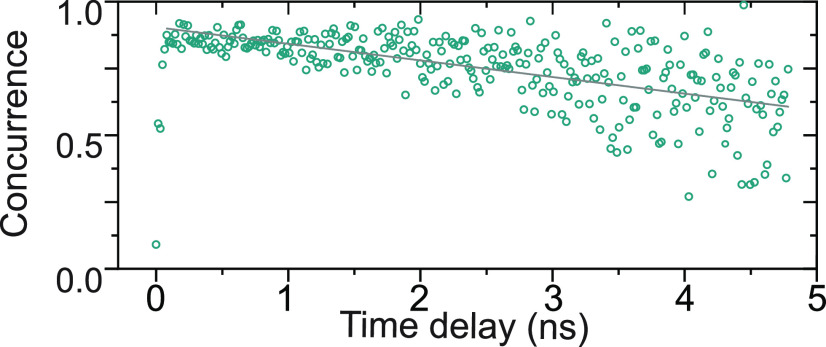

Entangled photons are an integral part in quantum optics experiments and a key resource in quantum imaging, quantum communication, and photonic quantum information processing. Making this resource available on-demand has been an ongoing scientific challenge with enormous progress in recent years. Of particular interest is the potential to transmit quantum information over long distances, making photons the only reliable flying qubit. Entangled photons at the telecom C-band could be directly launched into single-mode optical fibers, enabling worldwide quantum communication via existing telecommunication infrastructure. However, the on-demand generation of entangled photons at this desired wavelength window has been elusive. Here, we show a photon pair generation efficiency of 69.9 ± 3.6% in the telecom C-band by an InAs/GaAs semiconductor quantum dot on a metamorphic buffer layer. Using a robust phonon-assisted two-photon excitation scheme we measure a maximum concurrence of 91.4 ± 3.8% and a peak fidelity to the Φ^+^ state of 95.2 ± 1.1%, verifying on-demand generation of strongly entangled photon pairs and marking an important milestone for interfacing quantum light sources with our classical fiber networks.

Most photonic quantum technologies rely on entanglement to generate a quantum advantage compared to their classical counterparts. Preeminent examples are super sensitivity and super resolution,^1,2^ entanglement-enhanced microscopy,^3^ ghost imaging,^4^ photonic one-way quantum computing,^5,6^ and (entanglement-based) quantum key distribution.^7,8^ Specifically quantum communication requires the transmission of such entangled states over long distances, making it favorable to generate the entangled photons in the telecom C-band (1530–1565 nm). This would yield the lowest absorption losses in deployed fiber networks. Historically, probabilistic sources based on spontaneous processes, e.g., parametric downconversion or four-wave mixing, have dominated the field of fiber-based entanglement distribution^9−13^ and moreover satellite-based long-distance communication.^14^ In recent years semiconductor quantum dots have emerged as strong competitors due to their promise of deterministic qubit generation, based on their unrivaled emission of on-demand single photons^15−18^ and entangled photon pairs.^17−23^ These outstanding properties hinge on the emission of photons via the radiative biexciton–exciton cascade, emitting polarization-entangled photon pairs.^24^ Recently, photons emitted by a semiconductor quantum dot were employed in a heralded CNOT operation, generating Bell states outside of the typical cascaded operation.^25^

In the presence of asymmetry, the excitonic states exhibit a fine-structure splitting resulting in the time-evolving two-photon Bell state generated by the radiative cascade:^26^
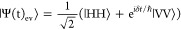
1Here, δ corresponds to the fine-structure splitting and ℏ to the reduced Planck’s constant. With appropriate time resolution the oscillating entangled state can be resolved and the measured degree of entanglement is unaltered.^21,27^

Using pulsed two-photon excitation of the biexciton state,^28^ giving rise to the desired cascaded emission, quantum dots achieved background-free emission of single photons,^15,16^ high degrees of entanglement with near-unity fidelity and concurrence,^22^ quantum teleportation,^29^ and entanglement swapping.^30,31^ These results underpin the great potential of the quantum dot biexciton–exciton cascade in quantum communication applications. However, all these achievements have been demonstrated outside of the telecom wavelength range. In the recent past, enormous progress in the fabrication of telecom quantum dots^32−34^ has been made, in particular the use of a metamorphic buffer layer to grow InAs quantum dots on GaAs substrates,^35,36^ having enabled the demonstration of entangled photons under nonresonant continuous-wave excitation.^37^ Here, we present on-demand generation of entangled photon pairs from epitaxially grown InAs quantum dots with a metamorphic buffer layer on a GaAs substrate, enabling emission in the telecom C-band (see Supporting Information and ref (35)).

## Telecom Entanglement Setup

**Figure 1:**
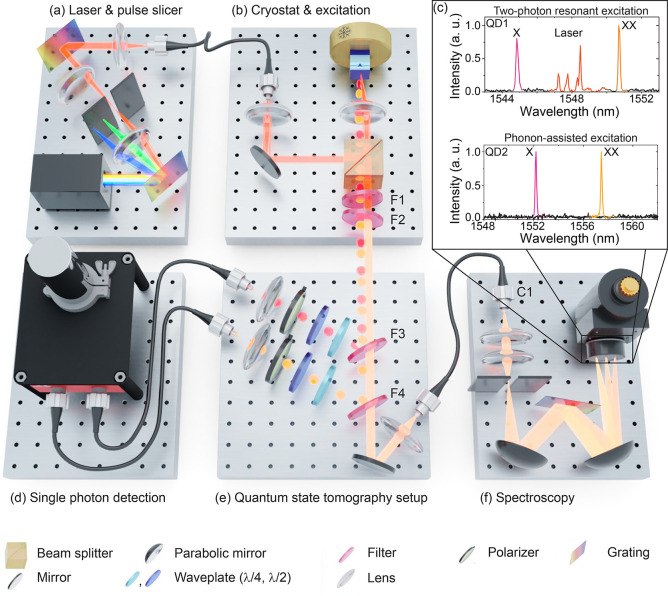
Telecom entanglement setup consisting of (a) laser excitation and pulse shaper, (b) cryogenically cooled InAs/GaAs quantum dot sample with excitation setup, (d) superconducting nanowire single-photon detectors for time-resolved measurements, (e) filtering and quantum state tomography setup, and (f) spectroscopy setup. (c) Quantum dot spectra for two-photon resonant excitation (top) and phonon-assisted two-photon resonant excitation (bottom). The TPE spectrum in (c) is recorded after filtering only with F1 and F2; the spectrum under phonon-assisted excitation in (c) is measured after the exciton (X) is reflected from F4 and the biexciton (XX) from F3, respectively.

The quantum dots are grown via metal–organic vapor-phase epitaxy, which is a scalable industry-grade growth method. The GaAs/InAs material system avoids the shortcomings of the InP-based emitters.^38^ A schematic illustration of the setup is presented in Figure 1, showing the excitation laser (a), cryostat and sample (b), single-photon detection (d), filtering with entanglement analysis (e), and spectroscopy setup (f). The sample is placed in a closed-cycle cryostat and cooled to 10 K (Figure 1(b)). To excite the sample, we use a tunable pulsed laser that generates 2 ps pulses with a repetition rate of 80 MHz. A pulse slicer is used to adjust the pulse length of the laser pulses between 2 and 70 ps (Figure 1(a)). After excitation, the emitted quantum dot photons are collected with a 0.8 NA objective and then sent to our spectrometer (Figure 1(b) and (f)) and entanglement analysis setup (Figure 1(e)). Tunable notch filters (F) with a 0.7 nm spectral bandwidth can be used to either block the excitation laser (F1 and F2) or reflect a selected quantum dot transition and separate XX from X and from the remaining laser light (F3 and F4). F3 and F4 can be tuned such that the entire quantum dot spectrum can be sent to a spectrometer equipped with an InGaAs array for spectral analysis (typical resolution is 25 μeV). If tuned to the exciton and biexciton wavelengths, respectively, F3 and F4 deflect the quantum dot photon toward standard telecom single-mode fibers. The fibers carrying the XX or X photons can be connected to C1 for spectral analysis. A set of waveplates, as well as a polarizer in front of each fiber, are used to set the polarization basis for the quantum state tomography measurements. The fiber-coupled quantum dot photons are then connected to superconducting nanowire single-photon detectors with a time resolution of approximately 30 ps and efficiencies of 15% and 25% measured at a dark count level of 30 s^–1^ (Figure 1(d)).

## Photoluminescence Measurements

In the top part of Figure 1(c) we show a quantum dot spectrum in the telecom C-band recorded under two-photon resonant excitation (QD1). Excitonic and biexcitonic emission are visible in the spectrum at emission wavelengths of 1544.8 and 1550.7 nm, respectively. In between the two emission lines, scattered laser light is visible (red). For excitation laser pulses with a central wavelength of 1547.8 nm, a pulse length of 47 ps was used. In the bottom part of Figure 1(c) we show the spectra for exciton and biexciton of QD2 using the phonon-assisted two-photon excitation scheme. The spectra are recorded through optical fibers after the tunable filters F3 and F4 in addition to tunable fiber-based filters (not shown, 0.8 nm spectral bandwidth) tuned to the emission wavelength of either the exciton or the biexciton. For excitation we use 58 ps pulses with a center wavelength of 1554.8 nm. As clearly visible in the spectrum, the remaining contribution of the excitation laser after careful filtering is negligible. For the spectrum under phonon-assisted excitation (Figure 1(c), bottom panel), three filters per quantum dot transition have been used to suppress the laser, as opposed to only F1 and F2 in the case of TPE (spectrum in Figure 1(c), top panel), yielding a stronger laser suppression.

## Two-Photon Excitation Schemes

**Figure 2:**
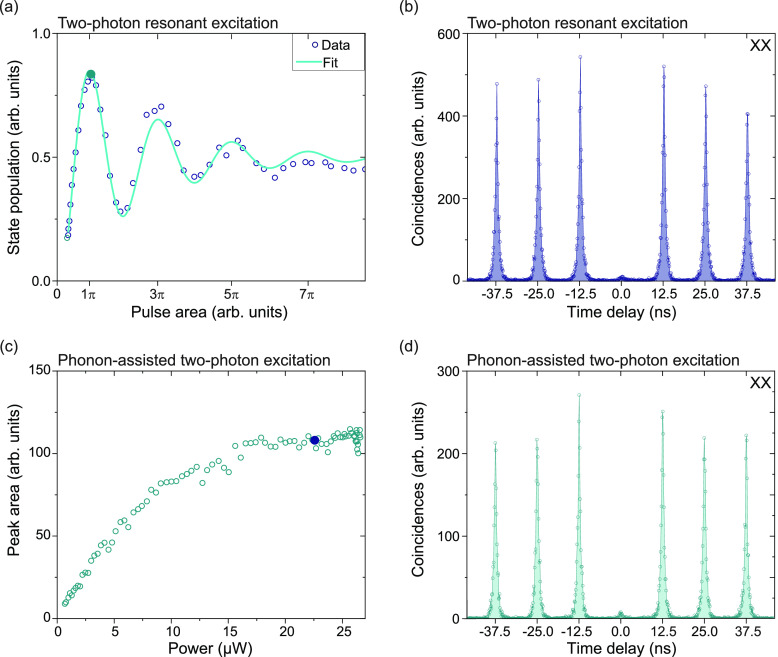
Two-photon resonant excitation in comparison with phonon-assisted excitation. (a) Excitation power-dependent peak area of the biexciton state of QD1 showing Rabi oscillations up to 7π. The filled dot represents the pulse area used to measure the autocorrelation. (b) Autocorrelation measurement of the biexciton state under π-pulse excitation. (c) Excitation power-dependent peak area of the exciton state of QD2 showing plateau-like saturation behavior for higher excitation powers. The filled dot represents the pulse area used to perform all following measurements. (d) Autocorrelation measurement of the biexciton state.

We show coherent control of the three-level system of QD1 via measuring the integrated peak area as a function of excitation power shown in Figure 2(a) for the biexciton exhibiting Rabi oscillations up to 7π. With a fit to the data we can extract the population in the π-pulse of 82.6 ± 1.6% (84.6 ± 2.7%) for the biexciton (exciton), yielding a photon pair generation efficiency of 69.9 ± 3.6%. Here, we estimate the pair generation efficiency as the product of the probabilities of the biexciton decayed with subsequent decay of the exciton state within the same excitation laser excitation cycle. For more details on the fit model, see Supporting Information. To stabilize the charge environment, we add approximately 100 nW of a continuous-wave laser with λ = 632.8 nm to our pulsed excitation laser. To determine the multiphoton suppression, we perform a second-order autocorrelation measurement of the biexciton under two-photon resonant π-pulse excitation, which is shown in Figure 2(b), yielding *g*^(2)^(0) = 0.043 ± 0.004 (0.074 ± 0.004 for the exciton, see Supporting Information). The degree of second-order coherence is determined by the ratio of peak areas between the center and the averaged side peaks by summing up all histogram events within the repetition period. The filtering in this case is performed by three filters (F1, F2, and F4 for the biexciton). Polarization-dependent photoluminescence measurements reveal a fine-structure splitting of 25 ± 7 μeV for this particular quantum dot (QD1). This causes a precession of the eigenstate with a period of  = 170 ps, making this quantum dot unsuitable for entanglement measurements with our setup time resolution (73 ps).

We would like to note that this excitation method requires some sort of stabilization to maintain the high biexciton state population. Besides demanding quantum dot specific excitation conditions, small fluctuations in excitation laser power or wavelength would cause diminished state occupation for the quantum dot. However, practical quantum networks relying on the interaction of multiple remote sources require excitation techniques that function for a variety of quantum dots and independent of environmental fluctuations. A more robust and universal scheme compared to pure two-photon excitation is phonon-assisted two-photon excitation.^39^ It enables the preparation of the excited state with close to maximum probability for a much broader range of excitation powers and wavelengths, while keeping consistently high levels of fidelities and indistinguishability of the prepared photons,^40^ making the scheme highly relevant for future applications that require several remote emitters at the same wavelength such as quantum teleportation or entanglement swapping. A power-dependent measurement of QD2 under phonon-assisted resonant excitation yields the curve shown in Figure 2(c), similar to the one previously reported in ref (40) for quantum dots emitting at 780 nm. All following measurements are taken with excitation powers well within the plateau at the end of the power-series curve. We also conduct a second-order autocorrelation measurement for QD2 under phonon-assisted excitation, which yields *g*^(2)^(0) = 0.038 ± 0.005 (0.068 ± 0.004 for the exciton), similar to the case of the pure two-photon resonant excitation used for QD1. From the time-tagged autocorrelation measurement, a biexciton lifetime of 446 ± 4 ps is extracted (see also Supporting Information). For the exciton we determine a lifetime of 1256 ± 50 ps.

## Generation of Highly Entangled Photons

**Figure 3:**
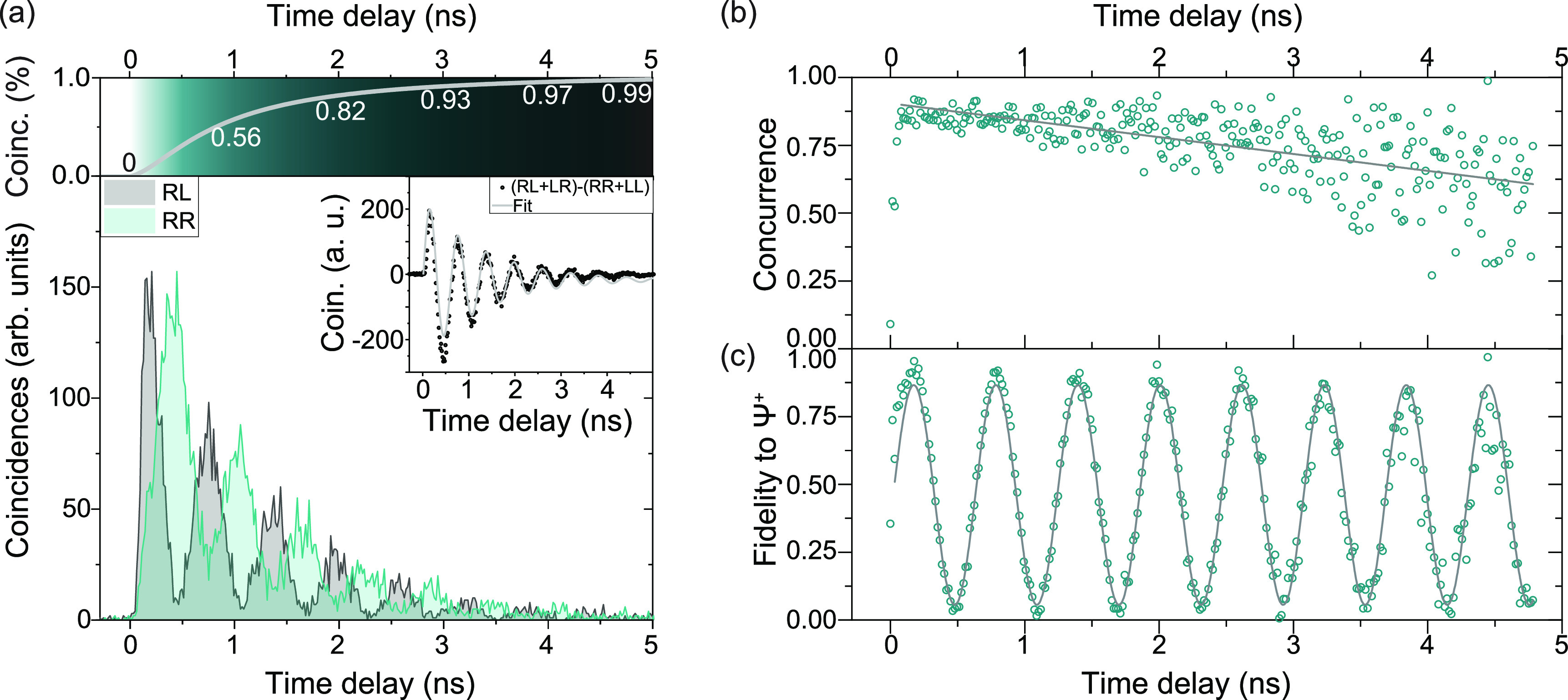
Results of quantum state tomography in the telecom C-band. (a) Top panel: Amount of total coincidences as a function of time. Bottom panel: Center peaks of two coincidence measurements recorded in the circular basis (RR and RL) that are showing oscillations due to the fine-structure splitting of QD2. Inset: Quantum oscillations between the two Bell states Φ^+^ and Φ^–^. (b) Concurrence reconstructed from the quantum state tomography measurements. The green open circles correspond to data; the gray solid line corresponds to a linear fit to the data. The maximum concurrence is 91.4 ± 3.8% for a time delay of 176 ps. (c) Fidelity to Φ^+^ as a function of time, showing oscillations due to the fine-structure splitting of QD2. The green open circles correspond to data; the gray solid line corresponds to a sine fit to the data.

**Figure 4:**
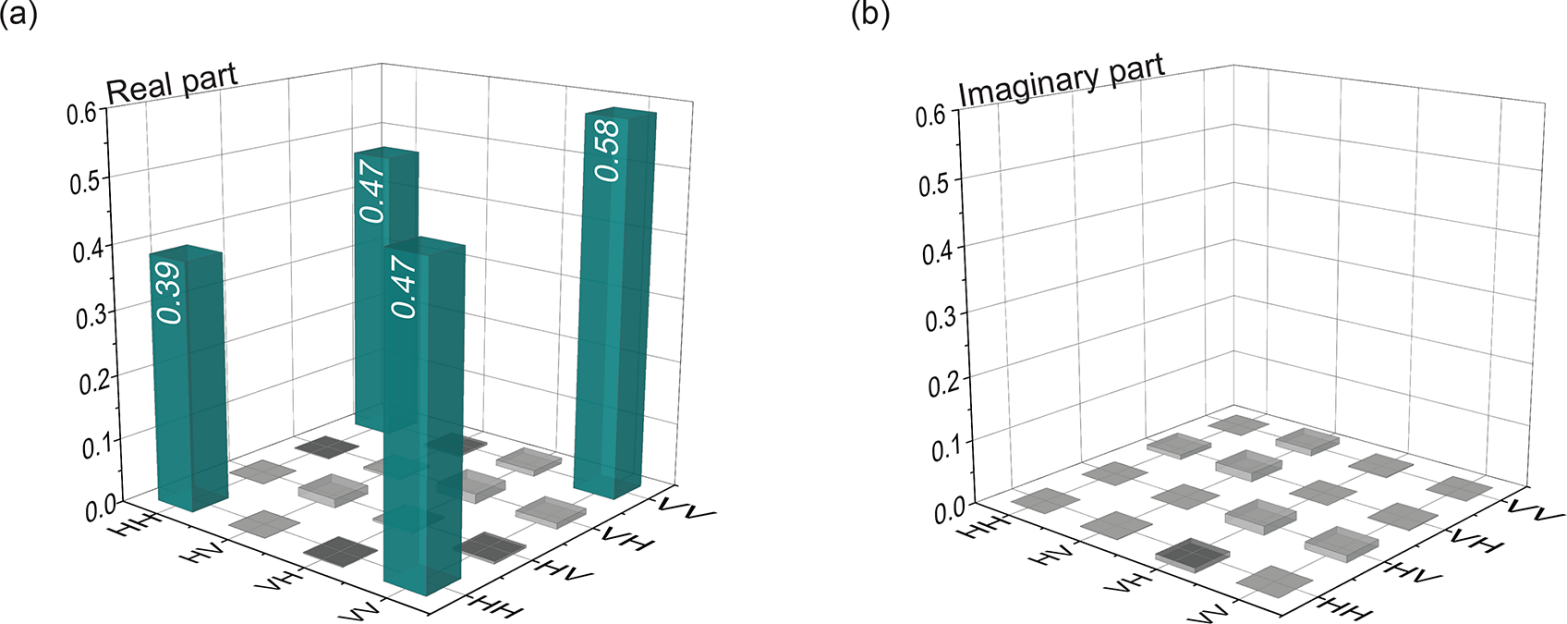
Density matrix of QD2 excited via phonon-assisted two-photon excitation. Real (a) and imaginary (b) part of the density matrix reconstructed for a time delay of 176 ps and a bin width of 16 ps.

To demonstrate that we can extract entangled photon pairs under phonon-assisted two-photon resonant excitation, we perform quantum state tomography on the two photon state generated by the XX–X cascade by recording time-tagged data in all 36 different polarization bases,^41^ from which we extract correlation histograms with our extensible time tag analyzing software.^42^ The center peak in the coincidence histogram shows oscillations (Figure 3(a)) due to the evolving polarization state of the exciton in the circular basis. The emitted photon state temporally oscillates between the two Bell states Φ^+^ = 1√2(|HH⟩ + |VV⟩) = 1√2(|RL⟩ + |LR⟩) and Φ^–^ = 1√2(|HH⟩ – |VV⟩) = 1√2(|RR⟩ + |LL⟩), which is shown in the inset of Figure 3(a). From the oscillations we can extract a value of the fine-structure splitting of 4.11 ± 0.13 μeV, yielding a more precise value due to higher resolution in the time domain compared to spectroscopy. To analyze the degree of entanglement, we extract the concurrence from our data using a modified version of the quantum state tomography code of ref (43). In Figure 3(a), we show the zero time delay peak for polarization settings RL and RR up to a time delay of 5 ns. 99% of correlation events happen within this time window (see top panel of Figure 3(a)). The concurrence, shown in Figure 3(b), stays well above 0.5 for all time bins evaluated in the entire 5 ns range, where a value of above 0 confirms the presence of entangled photons. Furthermore, we extract a maximum raw concurrence of 91.4 ± 3.8%, without correcting for the nonzero *g*^(2)^(0), the detector dark counts, or uncorrelated background light. This result is possible (i) due to the high time resolution of our detection system, which allows us to clearly observe the evolving state, and (ii) as a result of our excitation scheme, which directly populates the biexciton and strongly suppresses re-excitation within the same excitation pulse. Re-excitation would degrade the measurable entanglement even for quantum dots without fine-structure splitting. By averaging the concurrence weighted with the amount of coincidences per bin, we find a mean concurrence of 80.95 ± 0.47% (see also Supporting Information). This highlights the entanglement quality provided by the used excitation scheme and demonstrates that the decrease in concurrence in Figure 3(b) is only due to the increasing noise level at the tail of the quantum dot decay and not due to dephasing. Figure 3(c) shows the fidelity to the state  as a function of time. Also here the characteristic oscillations due to the fine-structure splitting of the quantum dot can be observed, making the emitted state oscillate between Φ^+^ and Φ^–^. The maximum raw fidelity to Φ^+^ is 95.2 ± 1.1% for a time delay of 176 ps, corresponding to a quantum bit error rate as low as 3.2% (calculated according to the Supporting Information of ref (44)). Finally, we reconstruct the density matrix from the quantum state tomography measurement following ref (41), which is presented in Figure 4 for a time delay of 176 ps and a bin width of 16 ps after it has undergone a coordinate transformation to compensate for birefringence caused by the sample, the cryostat, and the excitation part of the setup; see Figure 1(b). These elements perform a coordinate transformation of the photons that are originally emitted in the standard HV basis, to the birefringent H̃Ṽ basis. A virtual waveplate is introduced in order to transform back from the birefringent H̃Ṽ coordinate system to the HV basis in which the polarization analysis is performed. The applied transformation is preserving the orthogonality of the polarization basis such that the absolute values of, for example, the fidelity of a state in a given basis compared to a maximally entangled state are not changed. The maximally entangled state in the birefringent H̃Ṽ basis and the closest entangled state in the HV basis are given in the Supporting Information. The real part of the density matrix (Figure 4(a)) exhibits dominant outer diagonal elements, while all other elements of the matrix are strongly suppressed, with a negligible imaginary part (Figure 4(b)). This further highlights the unrivaled quality of the entangled photons created from our source via the phonon-assisted excitation scheme.

## Conclusion

We have demonstrated on-demand emission of polarization entangled photon pairs from an InAs/GaAs quantum dot in the telecom C-band. Besides the small fine-structure splitting, we are able to measure a maximum concurrence of up to 91.4 ± 3.8%. An unprecedented level of fidelity in the telecom C-band to Φ^+^ of 95.2 ± 1.1% was extracted without correcting for detector dark counts, background photons, or the nonzero *g*^(2)^(0) value. The high-quality entanglement that we are generating with our source is based on the state preparation via the phonon-assisted two-photon excitation scheme, combined with a good enough time resolution of our detection system compared to the fine-structure splitting of our quantum dot. The biexciton lifetime extracted under phonon-assisted excitation is significantly shorter than previously predicted by nonresonant excitation methods and demonstrates that these InAs/GaAs quantum dots could be operated at rates above the usual 80 MHz, allowing high quantum key rates. The industry-grade growth technique of the used quantum dots allows for wide availability of entangled photon emitters in the telecom C-band in the future and is, thus, a promising candidate for providing feasible sources for deployment in fiber-based quantum networks. Moreover, within the GaAs platform mature fabrication techniques for high-performance nanostructures with broadband enhancement efficiencies have been established,^17,18,45,46^ permitting high extraction efficiencies of on-demand entangled photon pairs in the telecom C-band from future devices. The high level of concurrence in combination with the resilient phonon-assisted excitation scheme has strong potential for any application relying on remote sources of entangled photons. Additional advances in growth techniques^47^ or integration onto a six-legged piezo device^48^ will enable a reduction and control of the fine-structure splitting. A combination structure with higher extraction efficiency and post-growth control over the quantum dot properties such as fine-structure splitting and emission energy will facilitate the selection of suitable quantum dots. The indistinguishability of photons generated by telecom C-band quantum dots should be determined in future experiments; however it would require s-shell resonant excitation to not be limited by the entanglement present in the cascaded emission.^49^ Furthermore, the on-demand generation of entangled photons opens up the possibility to transmit quantum secure keys efficiently over long distances, marking a step toward practical applications of quantum dots in fiber-based quantum networks.

## Supporting Information Available

Sample growth, quantum dot characterization, Rabi oscillations of QD1, Rabi oscillations of QD2, lifetimes of QD2, exciton autocorrelation, tomography setup characterization, two-photon state reconstruction, concurrence (PDF)
