# Supplementary material for: On-Demand Generation of Entangled Photon Pairs in the Telecom C-Band with InAs Quantum Dots
Source: ACS Photonics. 2021 Jul 15;8(8):2337–44. doi: 10.1021/acsphotonics.1c00504 (PMC8377713; doi:10.1021/acsphotonics.1c00504)
Supplement: Supplementary file 1 — ph1c00504_si_001.pdf [file ph1c00504_si_001.pdf]

Supporting information for 'On–demand generation of entangled photon pairs in the telecom C–band with InAs quantum dots'

Katharina D. Zeuner,[*,†] Klaus D. Jöns,[*,†] Lucas Schweickert,[†] Carl Reuterskiöld Hedlund,[‡] Carlos Nuñez Lobato,[‡] Thomas Lettner,[†] Kai Wang,[†] Samuel Gyger,[†] Eva Schöll,[†] Stephan Steinhauer,[†] Mattias Hammar,[‡] and Val Zwiller[†]

†*Department of Applied Physics, Royal Institute of Technology, Albanova University Centre, Roslagstullsbacken 21, 106 91 Stockholm, Sweden*

‡*Department of Electrical Engineering, Royal Institute of Technology, Electrum 229, 164 40 Kista, Sweden*

E-mail: zeuner@kth.se; klausj@kth.se

List of Figures

S1 Micro–photoluminescence spectra and polarization dependent measurements for two quantum dots in the telecom C–band. (a) Above–band spectrum of QD1. (b) Polarization dependent measurement for QD1. (c) Above–band spectrum of QD2. (d) Polarization dependent measurement for QD2. . . . . S5

S2 Histogram of finestructure splitting measurement of the used sample. The graph is adapted from Zeuner, K. (2020). Semiconductor Quantum Optics at Telecom Wavelengths (PhD dissertation, KTH Royal Institute of Technology). S6

S3 Rabi oscillations under pure two-photon resonant excitation (a) and two-photon resonant excitation with added laser with $\lambda_L = 632.8\,\mathrm{nm}$ (b). S8

S4 Rabi oscillations of QD2 (a) with additional background from phonon-assisted excitation due to spectrally impure laser pulses, (b) with removed phonon-background. Open circles correspond to data extracted from the fitted peaks, the solid line is a fit to the data. S10

S5 Biexciton lifetimes extracted under (a) above–band excitation and (b) phonon–assisted two–photon resonant excitation. S10

S6 Autocorrelation of the excitons of (a) QD1 under two-photon resonant excitation and (b) QD2 under phonon–assisted two–photon excitation. S11

S7 Laser state tomography measured for vertical input polarization into our analysis setup, (a) real part and (b) imaginary part of the resulting density matrix.S11

S8 Density matrices of the raw data (a, real part) and (b, imaginary part) and of the compensated data (c, real part) and (d, imaginary part). S14

S9 Fidelities to Φ^+ for different measurement bases. The open circles represent the data, the bold solid lines correspond to fits to the data. (a) Fidelity of $\tilde{\Phi}_{\mathrm{meas}}$ to Φ^+ with fit (blue) and Φ_{meas} with fit (turquoise) (b) Fidelity of Φ_{comp} to Φ^+ with fit (green open circles) and $\tilde{\Phi}_{\mathrm{meas}}$ to Φ_{max} (gray stars). S15

S10 Concurrence as a function of coincidence percentage in the center peak. . . . S16

List of Tables

S1 Details of used layer structure and growth parameters for emission in the telecom C–band. S4

Sample growth

The sample was grown by metal–organic vapor–phase epitaxy (MOVPE) on Si–doped GaAs (001)–oriented substrates in an Aixtron 200/4 low-pressure (100 mbar) horizontal reactor with H_2 as carrier gas and trimethylgallium (TMGa), trimethylaluminium (TMAl), trimethylindium (TMIn), and arsine (AsH_3) as precursors. The epitaxial layer structure is given Table 1. The distributed Bragg reflector (DBR) and compositionally graded InGaAs metamorphic buffer layer (MMBL) were first grown at 670 °C (calibrated wafer surface temperature) after which the growth was stopped and the temperature was reduced to 515 °C for quantum dot growth. Next, a 10 s ripening step was used and the low–temperature part of the capping layer was grown. Finally, the temperature was increased to 670 °C and the structure was completed with the high–temperature part of the capping layer. A three–lambda cavity is formed between the DBR and the semiconductor–air interface with the MMBL and capping layer thicknesses chosen to optimize the extraction efficiency. The lattice relaxation of the MMBL layer allows for the growth of large QDs with an emission wavelength of around 1550 nm, which is significantly longer than what can be obtained from the coherent growth on the GaAs substrate (typically <1300 nm)[1]. In our previous work, using similar growth conditions, we estimated the QD density to be in the $1 \times 10^7\,\mathrm{cm}^{-2}$ range[2].

Table S1: Details of used layer structure and growth parameters for emission in the telecom C–band.

Layer	Material	Thickness	Growth temperature	Comment
Substrate	GaAs	350 µm		
DBR (x19.5)	AlAs/GaAs	134.4 nm/114.6 nm	670 °C	
MMBL	$In_xGa_{1-x}As$	1150 nm	670 °C	x=0.015-0.4
QD	InAs		515 °C	
Low T Cap	$In_xGa_{1-x}As$	10 nm	515 °C	x=0.3
High T Cap	$In_xGa_{1-x}As$	195 nm	670 °C	x=0.3

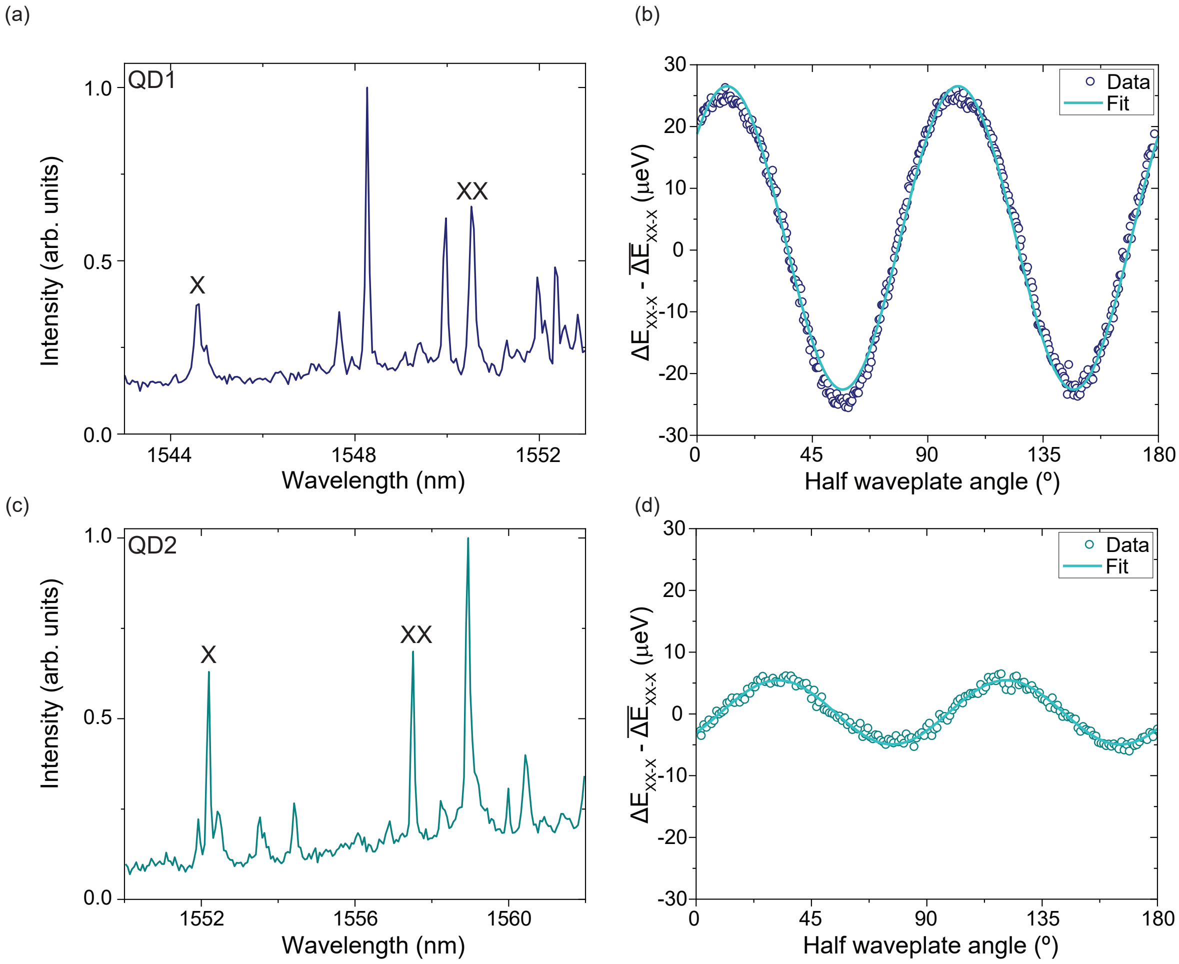

Figure S1: Micro–photoluminescence spectra and polarization dependent measurements for two quantum dots in the telecom C–band. (a) Above–band spectrum of QD1. (b) Polarization dependent measurement for QD1. (c) Above–band spectrum of QD2. (d) Polarization dependent measurement for QD2.

Quantum dot characterization

In Fig. S2 (a) and (c) spectra of quantum dot 1 and 2 are shown under above–band excitation with a continuous–wave laser with $\lambda_L = 632.8\,\text{nm}$. The exciton (X) and biexciton (XX) are labelled in the spectra, the other lines are attributed to charged transitions in the quantum dots, as they do not show polarization dependence. Both spectra are taken at saturation power. We would like to note that the background emission present for both quantum dots under above–band excitation is removed for two–photon resonant excitation and phonon–assisted TPE (shown in the main text). The oscillations of the emission energies of exciton

and biexction as a function of the detected linear polarization are shown in Fig. S2 (b) and (d). The data points are obtained via fits to spectra recorded for different linear polarization angles (open circles) and fitted with a sine function to extract the amplitude of the oscillation. We substract exciton and biexciton energies from each other to remove noise and eventual drifts of the setup during the measurement. QD1 exhibits a finestructure splitting of $\delta_1 = 25 \pm 7\,\mu\text{eV}$, which corresponds to a precession of the eigenstate with a period of $T = \frac{h}{\delta} = 170\,\text{ps}$, whereas QD2 exhibits $\delta_1 = 5 \pm 2\,\mu\text{eV}$, corresponding to a period of 800 ps. Within the error, this overlaps with the finestructure splitting calculated via the period of the quantum oscillations in the main text. We attribute the relatively large error bar in the polarization analysis to alignment imperfections of our setup. The errors are calculated via the fitting errors to each spectrum in our polarization dependent measurement. For the sample under investigation, we find a mean finestructure splitting of 6.0 μeV (7.6 μeV median) , with typical splittings as ranging between 2.6 μeV and 25 μeV. A histogram showing the distribution of finestructure values of the sample is shown in Figure S2.

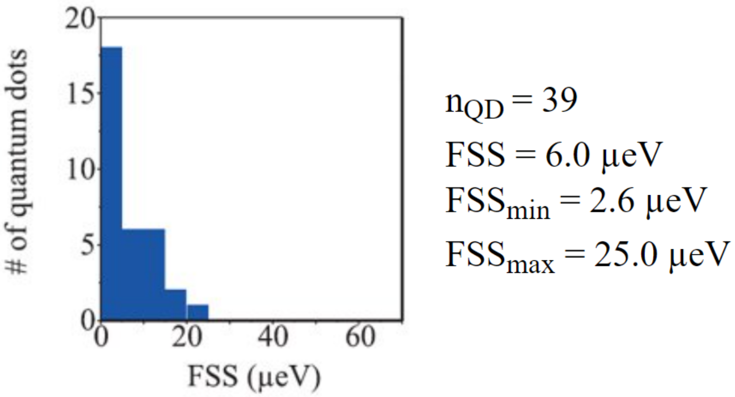

Figure S2: Histogram of finestructure splitting measurement of the used sample. The graph is adapted from Zeuner, K. (2020). Semiconductor Quantum Optics at Telecom Wavelengths (PhD dissertation, KTH Royal Institute of Technology).

Rabi oscillations of QD1

It has been reported in several articles in the literature that additional white light (or in our case above–band laser) can help to stabilize the charge environment[3,4] by saturating charges in the vicinity of the quantum dot. Unsaturated charges can in turn lead to fluctuating electric fields resulting in the quantum dot jumping in and out of resonance again and not yielding highest occupation probability in the π-pulse, visible in the power–dependence in Fig. S3 (a). We investigate the influence of the charge environment on the quantum dot state population and shape of the Rabi oscillations. We compare power–dependent measurements taken with only the pulsed excitation laser at the two–photon resonance (Fig. S3 (a) and with additional 100 nW of continuous–wave laser with $\lambda_L = 632.8\,\mathrm{nm}$ (Fig. S3 (b)). For our quantum dot, we see no effects on the shape of the Rabi oscillations when adding an order of magnitude less in continuous–wave laser power, but clear continuous–wave excitation when adding more than 1 µW. This observed behaviour emphasises the potential of the robust phonon–assisted excitation scheme which can prepare the highest state population independently of the charge environment without the presence of white light illumination or additional lasers. By adding low-powers of laser with $\lambda_L = 632.8\,\mathrm{nm}$, we observe the typical Rabi oscillations shown in Fig. S3 (b).

The population of the excited state is determined via the following fit to the data[5]:

$$c_2(t) = \frac{1}{2(1+2\chi^2)}\left(1 - \left(\cos\left(\Omega' t\right) + \frac{3\chi}{\sqrt{4-\chi^2}}\sin\left(\Omega' t\right)\right)\mathrm{e}^{-\frac{3\Gamma_1 t}{2}}\right). \tag{1}$$

Here, $c_2(t)$ is the probability amplitude to find the quantum system in the excited state. The following relations apply: $\chi = \Gamma_1/\Omega_0$, $\Omega' = \sqrt{\Omega_0^2 - \Gamma_1^2/4}$. Ω_0 is the Rabi frequency and Γ_1 corresponds to the decay rate of the excited state. $\Omega_0 = |-eE_0\mu_{12}/\hbar|$ is the Rabi frequency and E_0 the electrical field. The dipole matrix element μ_{12} is proportional to the excitation power $\Omega_0 \propto E_0 \propto \sqrt{P}$. This allows us to express the population of the excited

state depending on the pulse area via:

$$\Theta = \left| \frac{\mu_{12}}{\hbar} \int_{-\infty}^{\infty} E_0(t) dt \right|. \quad (2)$$

$\int_{-\infty}^{\infty} E_0(t)dt$ is the excitation pulse envelope. Via fits we obtain a population of $82.6 \pm 1.6\,\%$ for the biexciton and $84.6 \pm 2.7\,\%$ for the exciton. By multiplying these probabilities, we obtain a photon pair generation efficiency of $69.9 \pm 3.6\,\%$. Another way of determining the

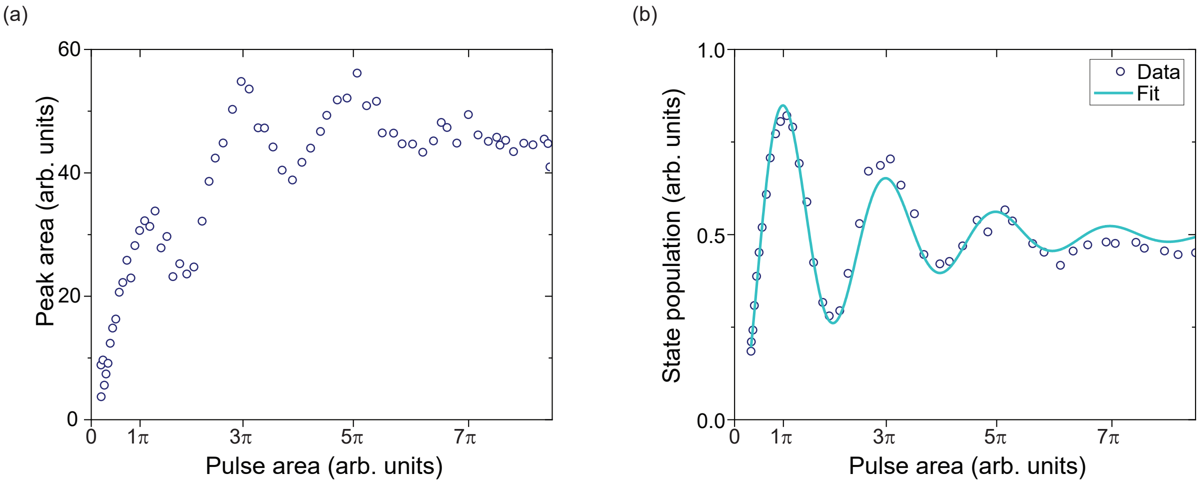

Figure S3: Rabi oscillations under pure two-photon resonant excitation (a) and two-photon resonant excitation with added laser with $\lambda_L = 632.8\,\mathrm{nm}$ (b).

photon pair generation efficiency is discussed in the supplementary material of reference[6]. Here, the authors suggest calculating the pair generation efficiency by comparing the peak areas of the center peak A_{center} in co–polarized cross correlation measurements (e.g. HH) and the corresponding side peak areas A_{side}. By taking the ratio of both quantities, the result is no longer dependent on the optical path efficiencies for biexciton and exciton. The pair generation efficiency p is related to the peak areas as follows:

$$\frac{\bar{A}_{center}}{\bar{A}_{side}} = \frac{2}{p} \quad (3)$$

Here, the $\bar{A}$ indicates that we average over all 6 co–polarized measurements. An excited-state preparation and radiation probability of $66.4 \pm 1.8\,\%$ is found, which is in good agreement

with the value obtained via fitting the Rabi oscillations.

Rabi oscillations of QD2

Power–dependent measurements for QD2 are shown in Fig. S4. During the measurement, we suffered from spectrally impure laser pulses after our pulse slicer, which did not allow us to perform pure two-photon resonant excitation. While we attempted to excite via the two–photon resonance, the effect due to the spectral profile of the laser can be described more as a two–photon excitation scheme with additional phonon contribution. As the laser pulses were not spectrally pure, there were additional spectral components partially detuned towards the phonon resonance energy. This results in the power–dependent measurement shown in Fig. S4 (a) that is not displaying the damped Rabi oscillations expected for two–photon resonant excitation. The additional and unwanted spectral components were visible in the spectrum and much broader than the bandwidth of our notch filters. This again highlights the robustness of the phonon–assisted scheme that is not relying on precise overlap of a laser spectrum with the quantum dot resonance. To be able to estimate the state population, we subtract the phonon background (see Fig. 2 (c) in the main text), obtaining the data points shown in Fig. S4 (b). A fit to the data yields a state population of $80.1 \pm 9.5\,\%$ in the π-pulse.

Lifetimes of QD2

We examine the influence of the excitation scheme on the radiative lifetimes of the biexciton of QD2. A decay time measurement under above–band excitation with 2 ps pulses at 1200 nm is shown in Fig. S5 (a). We find a decay time of $\tau_{above} = 794 \pm 3$ ps. The used fitting function is similar to the one in the supplementary material of Ref.[7] for the charged exciton. We would like to note that this is not the biexciton lifetime, as discussed in the appendix of Ref.[8]. In comparison, by using the phonon–assisted two–photon resonance excitation scheme the

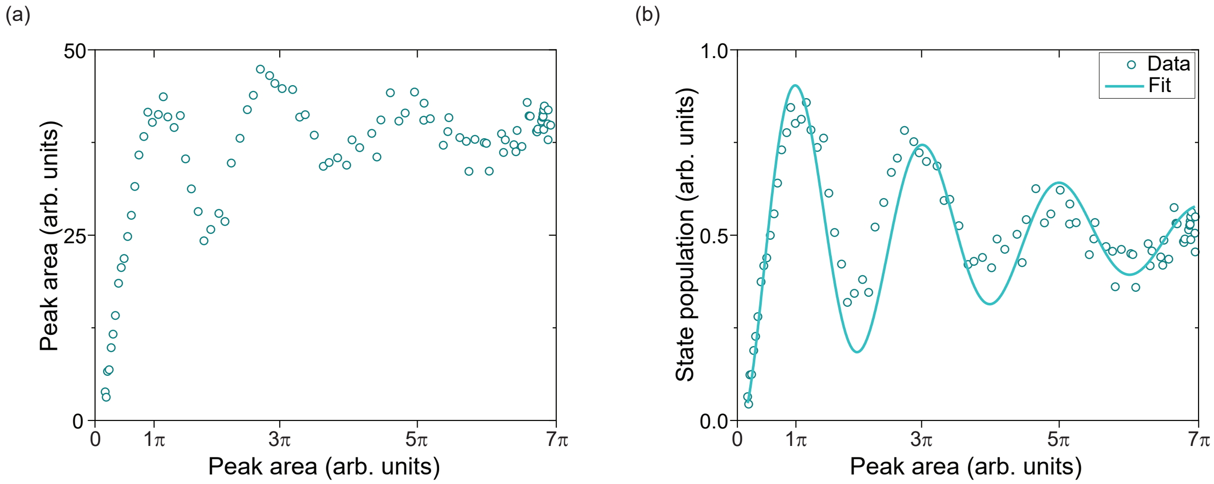

Figure S4: Rabi oscillations of QD2 (a) with additional background from phonon-assisted excitation due to spectrally impure laser pulses, (b) with removed phonon-background. Open circles correspond to data extracted from the fitted peaks, the solid line is a fit to the data.

lifetime extracted from the direct excitation of the biexciton is $\tau_{Phonon} = 446 \pm 4\,\text{ps}$. Pulsed resonant excitation schemes allow a more accurate extraction of lifetime, since there is no convolution of the actual radiative decay with the occupation processes happening for non–resonant excitation.

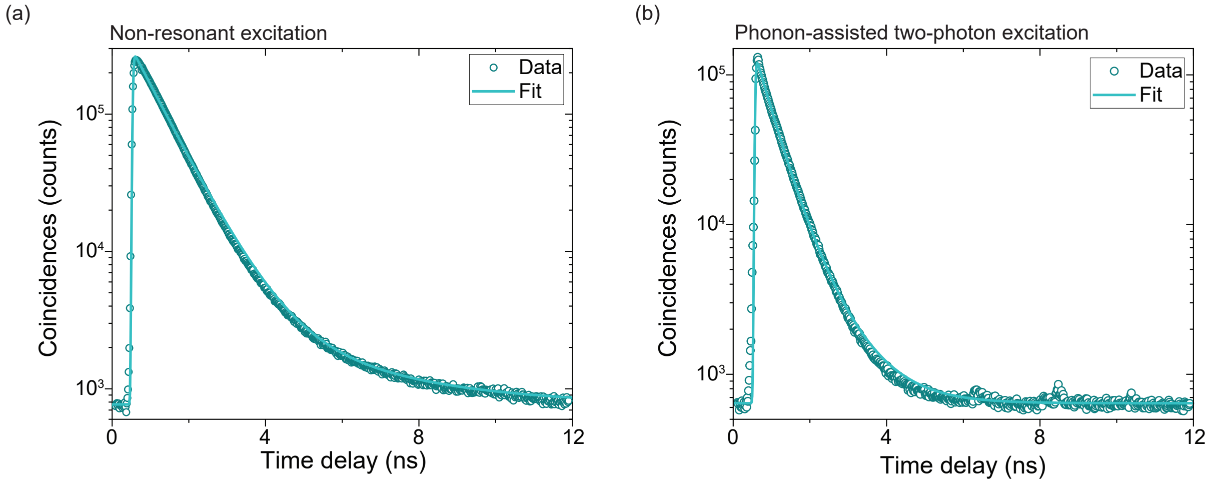

Figure S5: Biexciton lifetimes extracted under (a) above–band excitation and (b) phonon–assisted two–photon resonant excitation.

Exciton autocorrelation

In Figure S6 we show the autocorrelation measurements performed on the exciton of QD1 under two–photon resonant excitation in (a) and QD2 under phonon–assisted two–photon excitation in (b). We determine $g^{(2)}_{\mathrm{TPE}}(0) = 0.07 \pm 0.004$ for two–photon excitation and $g^{(2)}_{\mathrm{Phonon}}(0) = 0.068 \pm 0.004$ for phonon–assisted excitation.

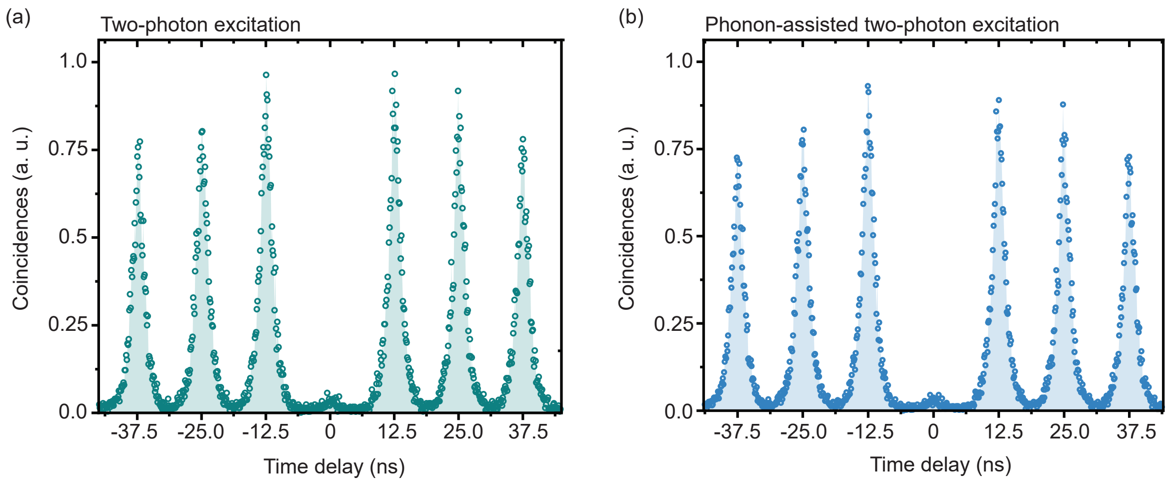

Figure S6: Autocorrelation of the excitons of (a) QD1 under two-photon resonant excitation and (b) QD2 under phonon–assisted two–photon excitation.

Tomography setup characterization

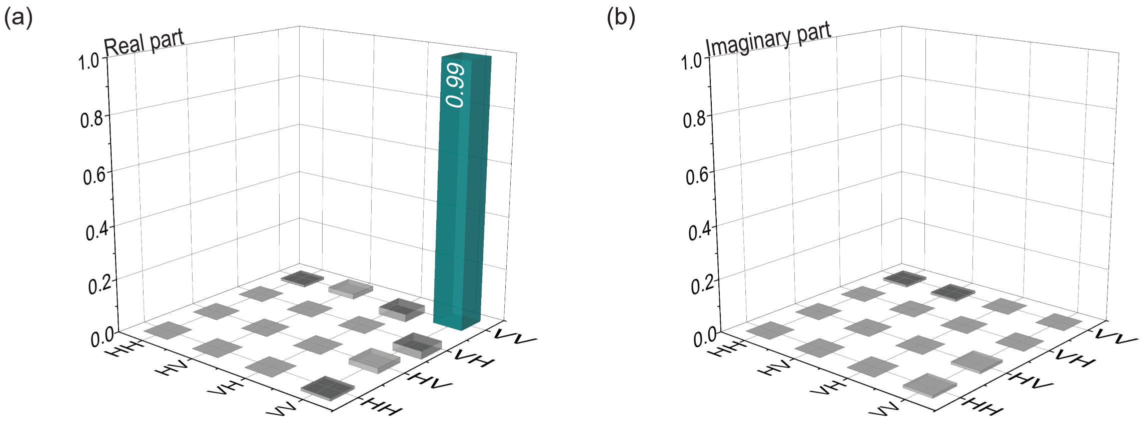

Figure S7: Laser state tomography measured for vertical input polarization into our analysis setup, (a) real part and (b) imaginary part of the resulting density matrix.

To make sure our quantum state tomography setup is carefully aligned, we perform a state tomography of the excitation laser, which is coupled into the analysis setup (see main text Fig. 1(e)), without passing the cryostat. The polarization of the laser is set to vertical using a half and a quarter waveplate, which is verified with a polarimeter. We perform all 36 measurements of the quantum state tomography with the vertically polarized laser, yielding the density matrix shown in Fig. S7 (a) and (b). From the matrix, we can infer that the waveplate angles are well calibrated, since there is only a peak for the $|\mathrm{VV}\rangle\langle\mathrm{VV}|$ state. The density matrix element of the vertical states amounts to 0.99, while the absolute values of all other elements are no larger than 0.03. From the imaginary part shown in Fig. S7 (b) we can infer that our analysis setup is not introducing significant additional phases.

Two–photon quantum state reconstruction

As mentioned in the main text, we perform a transformation from our birefringent measurement basis (birefringence introduced by the cryostat and sample) to the regular HV-coordinate base. The quantum dot photons are emitted in the HV–basis and we also perform the polarization analysis in this basis, but due to the setup birefringence the polarization of the photons are transformed to the $\tilde{\mathrm{H}}\tilde{\mathrm{V}}$–basis on the way from the cryostat to the entanglement analysis setup. The real and imaginary part of the raw density matrix for a time delay of 176 ps are shown in Fig. S8 (a) and (b), respectively. This matrix deviates from the expected matrix for an entangled state (Φ^+) with only elements in the corners and no contributions from other states, as well as a non–existing imaginary part. However, this is scrambled by the birefringence of our setup. Nevertheless, if the fidelity of this state in the birefringent basis to a maximally entangled state is calculated, this yields a fidelity of $95.23 \pm 1.90\,\%$. The corresponding state and density matrix are given below:

$$\tilde{\Phi}_{\mathrm{closest}} = (0.592 + 0\mathrm{i})\,|\tilde{\mathrm{H}}\tilde{\mathrm{H}}\rangle + (-0.100 + 0.211\mathrm{i})\,|\tilde{\mathrm{H}}\tilde{\mathrm{V}}\rangle$$

$$+ (-0.086 + 0.201\mathrm{i})\,|\tilde{\mathrm{V}}\tilde{\mathrm{H}}\rangle + (0.507 + 0.539\mathrm{i})\,|\tilde{\mathrm{V}}\tilde{\mathrm{V}}\rangle$$

$$\tilde{\rho}_{\mathrm{closest}} = \begin{pmatrix} 0.35 + 0\mathrm{i} & -0.05 - 0.11\mathrm{i} & -0.05 - 0.11\mathrm{i} & 0.29 - 0.31\mathrm{i} \\ -0.05 + 0.11\mathrm{i} & 0.07 + 0\mathrm{i} & 0.05 + 0.01\mathrm{i} & 0.06 + 0.16\mathrm{i} \\ -0.05 + 0.11\mathrm{i} & 0.05 - 0.01\mathrm{i} & 0.06 + 0\mathrm{i} & 0.06 + 0.14\mathrm{i} \\ 0.29 + 0.31\mathrm{i} & 0.06 - 0.16\mathrm{i} & 0.06 - 0.14\mathrm{i} & 0.53 + 0\mathrm{i} \end{pmatrix} \tag{4}$$

By applying a waveplate with the following properties (rotation $\theta = 0.352$ and phase $\phi = -0.516$), we transform the data back to the usual HV-basis, yielding a close to ideal density matrix for our entangled photons. The corresponding state and density matrix of the transformed state are:

$$\Phi_{\mathrm{transformed}} = (0.629 + 0\mathrm{i})\,|\mathrm{HH}\rangle + (-0.003 + 0.008\mathrm{i})\,|\mathrm{HV}\rangle$$

$$+ (-0.006 + 0.007\mathrm{i})\,|\mathrm{VH}\rangle + (0.777 + 0\mathrm{i})\,|\mathrm{VV}\rangle$$

$$\rho_{\mathrm{transformed}} = \begin{pmatrix} 0.39 + 0\mathrm{i} & 0 + 0\mathrm{i} & 0 + 0.01\mathrm{i} & 0.47 + 0\mathrm{i} \\ 0 - 0\mathrm{i} & 0.01 + 0\mathrm{i} & 0 + 0.01\mathrm{i} & -0.06 + 0.01\mathrm{i} \\ 0 - 0\mathrm{i} & 0.01 - 0\mathrm{i} & 0 + 0.01\mathrm{i} & -0.01 + 0.01\mathrm{i} \\ 0.47 - 0\mathrm{i} & -0.01 - 0.01\mathrm{i} & 0.01 - 0\mathrm{i} & 0.58 - 0\mathrm{i} \end{pmatrix} \tag{5}$$

The applied transformation is keeping the orthogonality of the polarizations. Furthermore, the fidelity of our state to $|\tilde{\mathrm{H}}\tilde{\mathrm{H}}\rangle + |\tilde{\mathrm{V}}\tilde{\mathrm{V}}\rangle$ in the $\tilde{\mathrm{H}}\tilde{\mathrm{V}}$-basis is the same as the fidelity to $|\mathrm{HH}\rangle + |\mathrm{VV}\rangle$ in the HV-coordinate system. In Fig. S9 (a) we show how the fidelity to Φ^+ is evolving over time for different initial states. The fidelity of the state $\tilde{\Phi}_{meas}$ in the birefringent coordinate system is shown as a function of time in Fig. S9 in blue. This yields a maximum fidelity of 71.3 % to Φ^+ because of the different polarization basis of $\tilde{\Phi}_{\mathrm{meas}}$ and Φ^+. The oscillation between the maximum fidelity and almost zero stems from

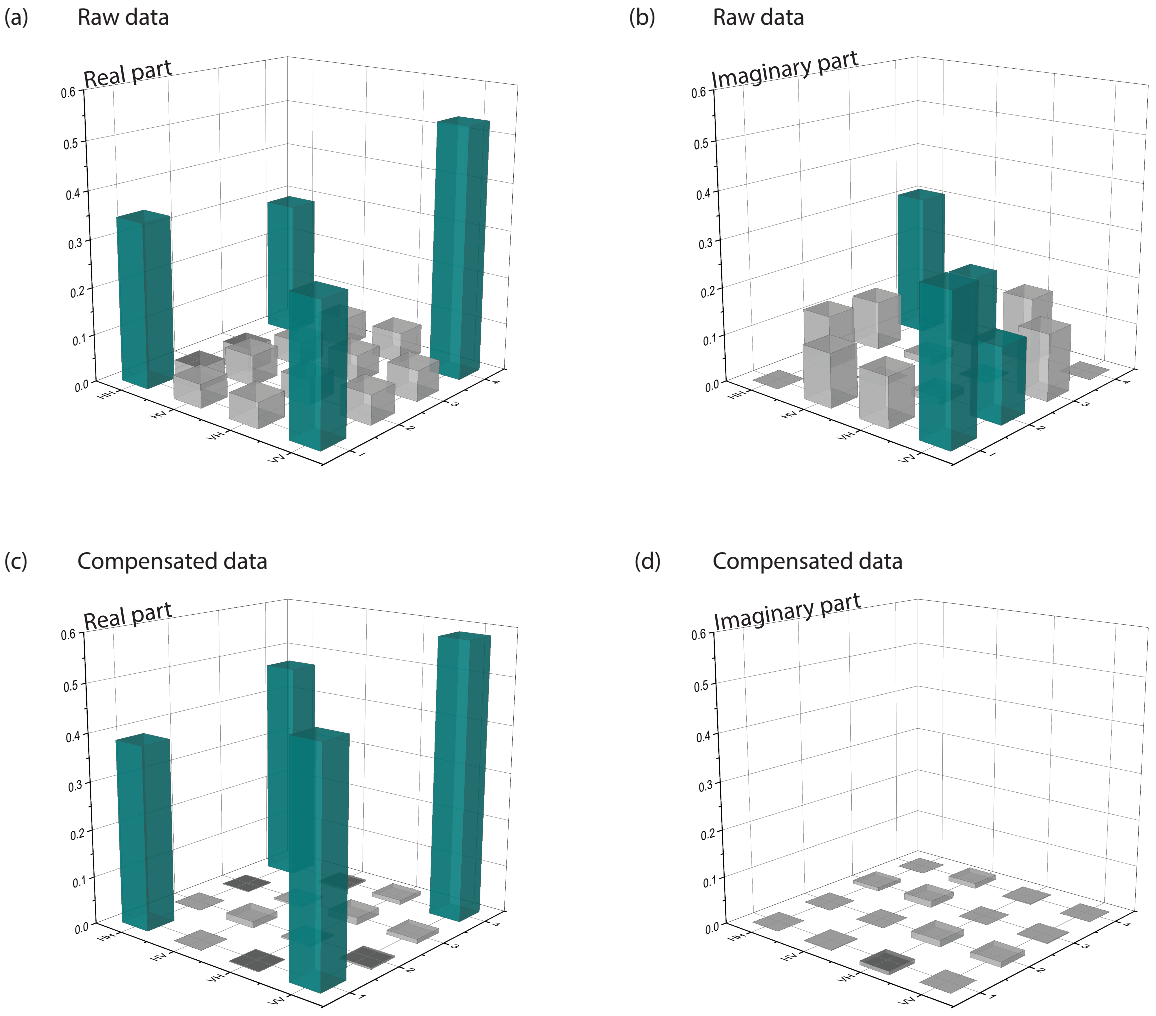

Figure S8: Density matrices of the raw data (a, real part) and (b, imaginary part) and of the compensated data (c, real part) and (d, imaginary part).

the oscillation of the emitted quantum state between Φ^+ and Φ^- due to the finestructure splitting. After applying the transformation to the HV-coordinate system, the maximum fidelity of our state compared to Φ^+ is increased to $95.2 \pm 1.1\,\%$ shown in turquoise. Now an almost perfect visibility in the oscillation between the two states is achieved. Finally, we calculate the fidelity of the measured state to Φ^+ after applying an optimal waveplate to each individual time bin compensating for the FSS. This yields a nearly flat fidelity to Φ^+, with a maximum fidelity of $95.4\,\%$, which is shown in Fig. S9 (b) with green open circles. In comparison, we plot the fidelity of $\tilde{\Phi}_{meas}$ to a maximally entangled state in the $\tilde{\mathrm{H}}\tilde{\mathrm{V}}$–basis,

showing full overlap within measurement uncertainty.

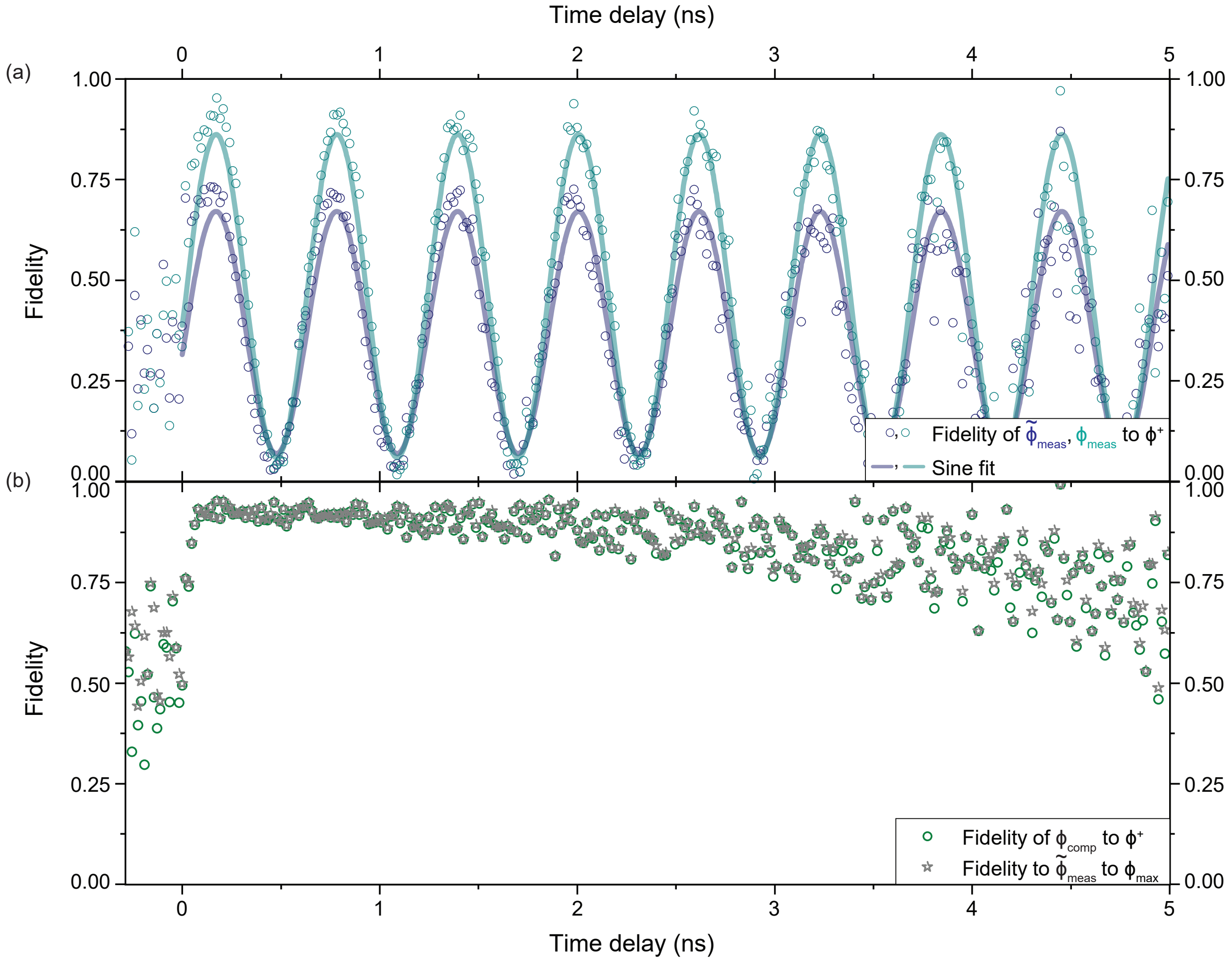

Figure S9: Fidelities to Φ^+ for different measurement bases. The open circles represent the data, the bold solid lines correspond to fits to the data. (a) Fidelity of $\tilde{\Phi}_{\mathrm{meas}}$ to Φ^+ with fit (blue) and Φ_{meas} with fit (turquoise) (b) Fidelity of Φ_{comp} to Φ^+ with fit (green open circles) and $\tilde{\Phi}_{\mathrm{meas}}$ to Φ_{max} (gray stars).

Concurrence

To demonstrate that the concurrence is only decreasing over time due to an increased noise level for time delays larger than 3 ns, we add a different way of plotting the data. In Figure S10, we plot the concurrence not as a function of time but instead as a function of already detected coincidences of the center peak. To do so, we sum up all coincidences in the center peak and normalize to the total amount of coincidences. In this representation it is apparent

that the concurrence is only decreasing distinctly after more than 90 % of the coincidences in the center peak have been measured, emphasizing that the decrease is only related to the noise level at this point in the measurement and not dephasing effects.

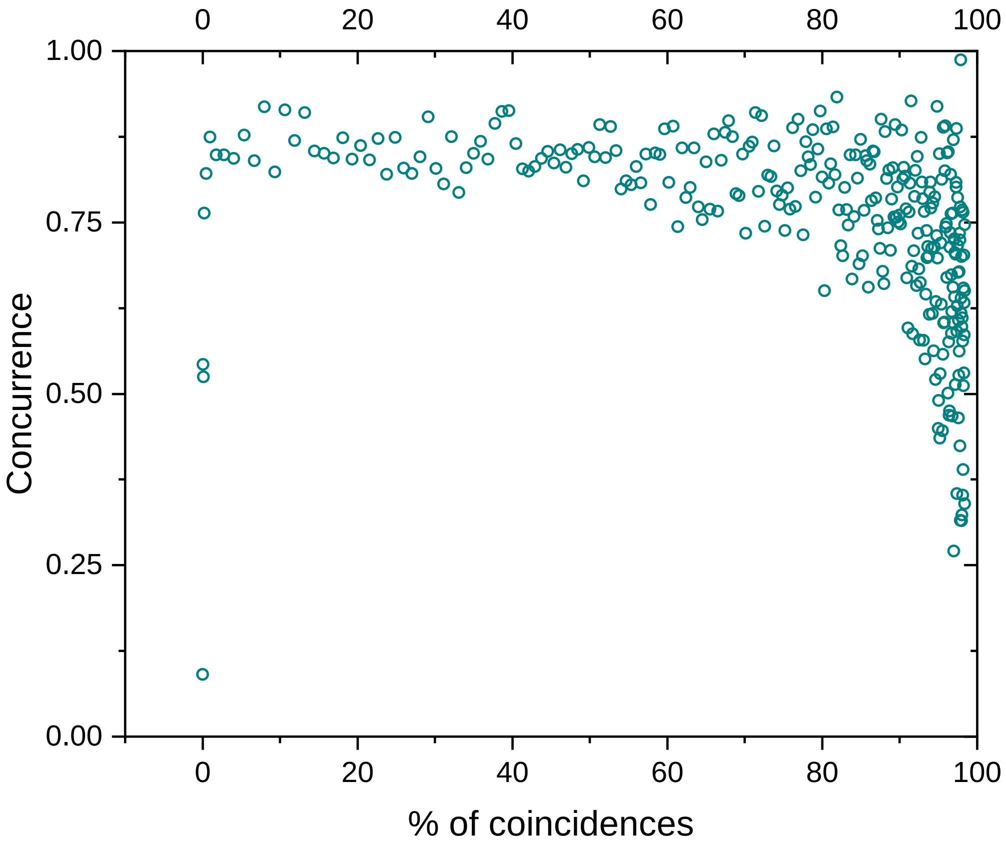

Figure S10: Concurrence as a function of coincidence percentage in the center peak.

References

(1) Paul, M.; Olbrich, F.; Höschele, J.; Schreier, S.; Kettler, J.; Portalupi, S. L.; Jetter, M.; Michler, P. Single-photon emission at 1.55 μ m from MOVPE-grown InAs quantum dots on InGaAs/GaAs metamorphic buffers. *Appl. Phys. Lett.* **2017**, *111*, 1–10.

(2) Zeuner, K. D.; Paul, M.; Lettner, T.; Reuterskiöld Hedlund, C.; Schweickert, L.; Stein-

hauer, S.; Yang, L.; Zichi, J.; Hammar, M.; Jöns, K. D.; Zwiller, V. A stable wavelength-tunable triggered source of single photons and cascaded photon pairs at the telecom C-band. *Appl. Phys. Lett.* **2018**, *112*, 173102.

(3) Gazzano, O.; Michaelis De Vasconcellos, S.; Arnold, C.; Nowak, A.; Galopin, E.; Sagnes, I.; Lanco, L.; Lemaître, A.; Senellart, P. Bright solid-state sources of indistinguishable single photons. *Nat. Commun.* **2013**, *4*, 1425.

(4) Reindl, M.; Jöns, K. D.; Huber, D.; Schimpf, C.; Huo, Y.; Zwiller, V.; Rastelli, A.; Trotta, R. Phonon-Assisted Two-Photon Interference from Remote Quantum Emitters. *Nano Lett.* **2017**, *17*, 4090–4095.

(5) Fox, M. *Quantum optics: an introduction*; Oxford master series in atomic, optical, and laser physics; Oxford Univ. Press: Oxford, 2006.

(6) Wang, H. et al. On-Demand Semiconductor Source of Entangled Photons Which Simultaneously Has High Fidelity, Efficiency, and Indistinguishability. *Phys. Rev. Lett.* **2019**, *122*, 1–6.

(7) Schöll, E.; Hanschke, L.; Schweickert, L.; Zeuner, K. D.; Reindl, M.; Filipe Covre da Silva, S.; Lettner, T.; Trotta, R.; Finley, J. J.; Muüller, K.; Rastelli, A.; Zwiller, V.; Jöns, K. D. Resonance Fluorescence of GaAs Quantum Dots with Near-Unity Photon Indistinguishability. *Nano Lett.* **2019**, *19*, 2404–2410.

(8) Jahn, J.-P.; Munsch, M.; Béguin, L.; Kuhlmann, A. V.; Renggli, M.; Huo, Y.; Ding, F.; Trotta, R.; Reindl, M.; Schmidt, O. G.; Rastelli, A.; Treutlein, P.; Warburton, R. J. An artificial Rb atom in a semiconductor with lifetime-limited linewidth. *Phys. Rev. B* **2015**, *92*, 245439.
